# Machine learning for emerging infectious disease field responses

**DOI:** 10.1038/s41598-021-03687-w

**Published:** 2022-01-10

**Authors:** Han-Yi Robert Chiu, Chun-Kai Hwang, Shey-Ying Chen, Fuh-Yuan Shih, Hsieh-Cheng Han, Chwan-Chuen King, John Reuben Gilbert, Cheng-Chung Fang, Yen-Jen Oyang

**Affiliations:** 1 Department of Emergency Medicine, National Taiwan University Hospital and College of Medicine, National Taiwan University, No. 7 Chung Shan S. Road, Taipei, 100 Taiwan, ROC; 2 Department of Computer Science and Information Engineering, National Taiwan University, Taipei, 106 Taiwan, ROC; 3 National Taiwan University Cancer Center, National Taiwan University, Taipei, 106 Taiwan, ROC; 4 Research Center for Applied Sciences, Academia Sinica, Taipei, 115 Taiwan, ROC; 5 Institute of Epidemiology and Preventive Medicine, College of Public Health, National Taiwan University, Taipei, 100 Taiwan, ROC; 6 Institute of Biomedical Electronics and Bioinformatics, College of Electrical Engineering and Computer Science, National Taiwan University, No. 1, Sec. 4, Roosevelt Road, Taipei, 106 Taiwan, ROC

**Keywords:** Infectious diseases, Health services, Public health, Risk factors, Biomedical engineering

## Abstract

Emerging infectious diseases (EIDs), including the latest COVID-19 pandemic, have emerged and raised global public health crises in recent decades. Without existing protective immunity, an EID may spread rapidly and cause mass casualties in a very short time. Therefore, it is imperative to identify cases with risk of disease progression for the optimized allocation of medical resources in case medical facilities are overwhelmed with a flood of patients. This study has aimed to cope with this challenge from the aspect of preventive medicine by exploiting machine learning technologies. The study has been based on 83,227 hospital admissions with influenza-like illness and we analysed the risk effects of 19 comorbidities along with age and gender for severe illness or mortality risk. The experimental results revealed that the decision rules derived from the machine learning based prediction models can provide valuable guidelines for the healthcare policy makers to develop an effective vaccination strategy. Furthermore, in case the healthcare facilities are overwhelmed by patients with EID, which frequently occurred in the recent COVID-19 pandemic, the frontline physicians can incorporate the proposed prediction models to triage patients suffering minor symptoms without laboratory tests, which may become scarce during an EID disaster. In conclusion, our study has demonstrated an effective approach to exploit machine learning technologies to cope with the challenges faced during the outbreak of an EID.

## Introduction

Emerging infectious diseases (EIDs), including the severe acute respiratory syndrome (SARS) (2003)^1^, H1N1 influenza virus (2009)^2^, Middle East respiratory syndrome coronavirus (MERS-CoV) (2012)^3^, and coronavirus disease 2019 (COVID-19) pandemic^4^, emerged and raised global public health crises in recent decades. Without existing protective immunity at both individual and population levels, an emerging infectious disease may spread efficiently and lead to massive severe cases and mortality in the community^5^. In particular, with a highly contagious novel respiratory infectious disease^6^, medical resources, including medications, personal protective and life-supporting equipment, may be quickly exhausted once hospitals are overwhelmed with infected patients^7,8^. It may inevitably cause excessive mortality as demonstrated in many countries during the 2020–2021 COVID-19 pandemic^8,9^. As the clinical spectrum of emerging respiratory infections may range from asymptomatic or mild respiratory symptoms to severe pneumonia or acute respiratory distress syndrome^10,11^, it is therefore imperative for first-line physicians to prioritize scarce medical resources for critically ill patients and early symptomatic patients with high risk of rapid progression and death^9,12^. However, in the early stage of the outbreak of a novel respiratory infectious disease, there is usually no prior knowledge and available guidelines for the physicians to optimize medical decisions. Accordingly, it is of interest to investigate how to exploit machine learning (ML) technologies to cope with this challenge.

In recent years, ML technologies have been widely exploited in medical and public health research^12–14^. ML algorithms are highly effective in analyzing interactions among multiple, complex variables in clinical databases and making accurate predictions, while it may take a medical practitioner months or even years to accumulate sufficient experience to develop a decision making process. However, there are a wide range of ML algorithms with very different characteristics and design goals. At one end of the spectrum, advanced ML algorithms such as the deep neural network (DNN)^14,15^ and the support vector machine (SVM)^16^ employ complicated non-linear transformations to achieve superior prediction accuracy. However, due to the complicated non-linear transformations involved, it is essentially impossible to figure out how these kinds of ML algorithms make predictions. At the other end of the spectrum, ML algorithms such as decision trees (DT)^17–19^ and the naïve Bayesian classifier follow highly interpretable decision processes to make predictions^20^ but may suffer inferior prediction accuracy due to lack of non-linear transformations involved in the prediction process. The trade-off between prediction accuracy and interpretability with alternative ML algorithms may be an everlasting dilemma depending on different clinical applications. As pointed out by Flaxman and Vos, for some applications, using an explainable approach is more understandable and favourable for physicians even when it results in a slight reduction in accuracy^20^.

As ML technologies have been widely exploited in medical and public health research, it is not surprising to observe that scientists have been developing ML-based prediction models to address the challenges faced in the recent COVID-19 pandemic^21–29^. Several prediction models have been proposed to identify those COVID-19 infected patients with a high risk of progression to severe diseases^26–28^ or even death^21–25^. These studies extracted hospital COVID-19 cohorts, which included clinical presentations, laboratory data, and even images, to predict the risk of severe diseases and fatality. In this study, we have aimed to address the challenges brought by an EID disaster from the aspect of preventive medicine. Accordingly, we have incorporated only age, sex, and comorbidities as features to build the ML based prediction models for identifying the population at risk of severe diseases before infection. The proposed ML models are of significant merit when health policymakers need to identify high risk populations and then develop a prioritized vaccination strategy accordingly. For this scenario, we have developed prediction models that can provide health policymakers with explicit decision rules. These decision rules can also be exploited to educate the people with high risk to seek medical treatments promptly once they develop symptoms. In the recent COVID-19 pandemic, almost all countries with community outbreaks experienced unprecedented mortality due to the collapse of their healthcare systems. In such a scenario, the frontline physicians could incorporate our proposed prediction models to triage patients without laboratory tests, which could become scarce during a pandemic, in order to discharge patients with minimal risk. In this study, we have developed three types of prediction models, namely, the DT models^30,31^, the state-of-the-art DNN models^15,29^, as well as the conventional logistic regression-based prediction models. We have further conducted comprehensive analyses on the performance delivered by different types of prediction models.

## Methods

### Data collection and outcome measurement

**Figure 1:**
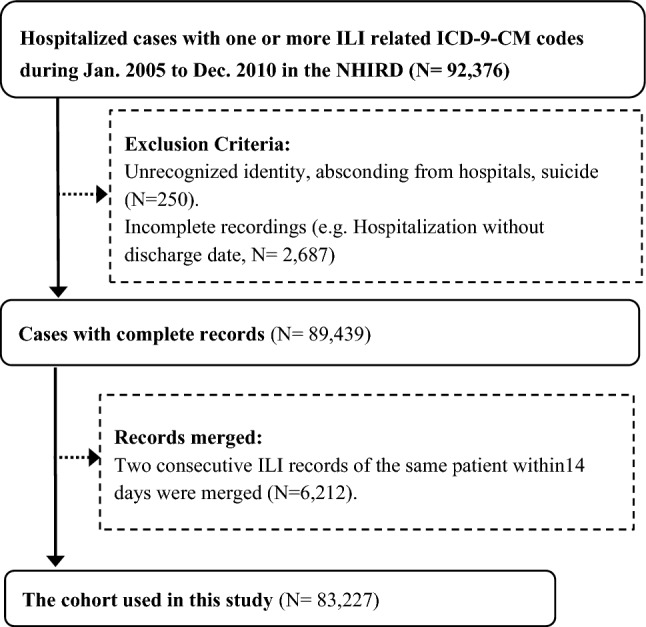
Flow diagram of the selection process of study subjects. *ICD-9-CM* International Classification of Diseases, 9th Revision, Clinical Modification, *ILI* influenza-like illness, *NHIRD* National Health Insurance Research Database.

We conducted this study based on the reimbursement data of one million randomly sampled subjects extracted from the de-identified National Health Insurance Research Database (NHIRD) in Taiwan. Figure 1 shows the process to generate the cohort. We began with 92,376 hospitalized ILI cases during January 2005 to December 2010. Supplementary Table S1 lists the ICD-9-CM (International Classification of Diseases, 9th Revision, Clinical Modification) codes employed to define an ILI case, which were identified through syndromic surveillance^32^ and intensive discussions among Taiwanese physicians^33,34^. The information retrieved from ILI patients’ records included age, gender, and 19 comorbidities/conditions [heart disease, peripheral vascular disease, hypertension, cerebrovascular accident (CVA), neurological disease, pulmonary disease, allergic rhinitis, autoimmune disease, liver disease, diabetes, hyperthyroidism, hypothyroidism, renal disease, metastatic cancer, cancer without metastasis, leukaemia/lymphoma, acquired immunodeficiency disease, tuberculosis, mental illness, and pregnancy/postpartum women]. These comorbidities were identified based on a literature review and thorough consensus reached by physicians of infection, emergency medicine, occupational health and infectious disease epidemiologists^35^. The corresponding ICD-9-CM codes employed to identify the 19 comorbidities are shown in Supplementary Table S2, which were defined based on the Charlson^36,37^, Deyo^38^ and Elixhauser^39^ measurements plus information from the Taiwanese Catastrophic Illness Card. Presence of a comorbidity was defined based on whether the patient was coded with the corresponding ICD-9-CM codes within 12 months prior to the index date of the ILI-related hospitalisation.

With the initial 92,376 hospitalized ILI cases, we excluded 250 cases with unrecognized identity, that left against medical advice from hospitals, or committed suicide and additional 2687 cases with incomplete records. Then, we merged two consecutive records of the same patient if these two consecutive records were within 14 days. In the end, a cohort containing 83,227 cases was created (Fig. 1). In the end, a cohort containing 83,227 cases was created (Fig. 1) and the demographic analysis of the cohort is presented in Supplementary Table S3.

The outcome of concern was severe ILI, which was defined as the occurrence of fatality or requiring critical cares such as intubation, ventilator support, extracorporeal membrane oxygenation treatment, admission to an intensive care unit during the hospitalization period. The study was approved by the Research Ethics Committee of the National Taiwan University Hospital (ID: 201603086RINB, April 14, 2016), and was performed in accordance with the Declaration of Helsinki.

### Experimental procedures

**Figure 2:**
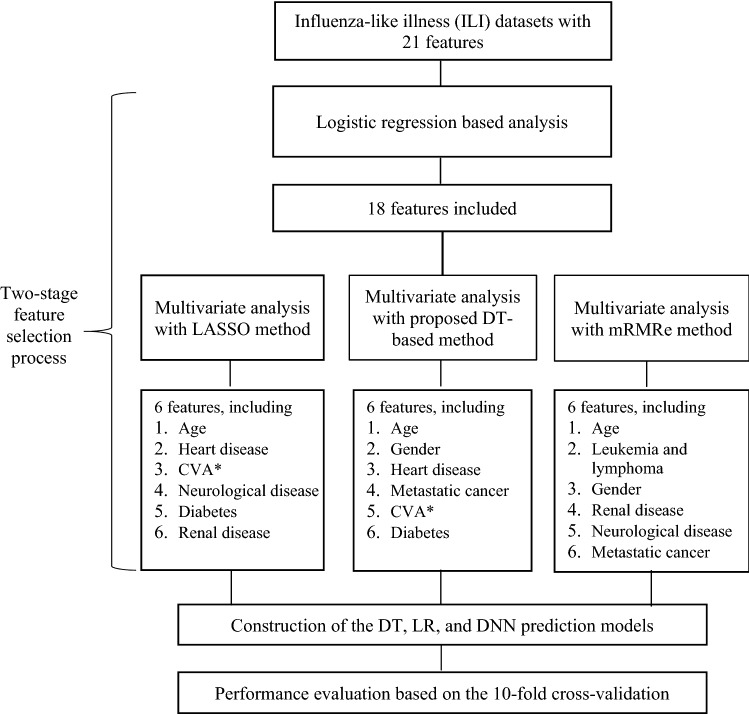
The experimental procedures. The analysis began with a 2-stage feature selection process. In the first stage, the conventional logistic regression analysis was employed to eliminate those features that were uncorrelated to the outcome variable. In the second stage, the proposed DT-based method along with two advanced multivariate analysis methods, namely being the least absolute shrinkage and selection operator (LASSO) method^40^ and the ensemble variant of minimum redundancy maximum relevance (mRMRe) method^41,42^, were employed to generate three 6-variable feature sets. Then, these three 6-variable feature sets were employed to build the DT, LR, and DNN prediction models. Finally, the performance of the alternative prediction models was evaluated based on the 10-fold cross validation process. *CVA cerebrovascular accident.

Figure 2 shows the experimental procedure employed in this study to analyze the performance delivered by different types of prediction models. The analysis began with a 2-stage feature selection process. In the first stage, we employed the conventional logistic regression (LR) analysis to eliminate those features that were uncorrelated to the outcome variable. Then, in the second stage, two advanced multivariate analysis methods, namely being the least absolute shrinkage and selection operator (LASSO) method^40^ and the ensemble variant of minimum redundancy maximum relevance (mRMRe) method^41,42^, were employed along with the proposed DT-based method to determine the minimal subsets of the features without compromising prediction performance. With the three feature sets output by the LASSO, the mRMRe, and the proposed DT-based method, we proceeded to build the DT^17–19^, the LR^43^, and the deep neural network (DNN)^15^ prediction models. Finally, the performance of these different prediction models were evaluated using 10-fold cross validation^44^.

### Feature selection

The feature selection process began with the 21-variable feature set shown in Table 1. Table 1 also shows the results of the first-stage LR based analysis. Since the p-values with mental illness, hypothyroidism, and hyperthyroidism were higher than 0.05, these three comorbidities were excluded. With the remaining 18 variables, we proceeded to carry out the DT-based multivariate analysis proposed in this study. In this procedure, the DT package^18^ shown in Supplementary Table S4 was employed and parameters prior, which specifies the prior priority of positive cases, and cp, which controls the complexity of the output tree, were set to different values in order to generate models with various sensitivity levels. Supplementary Table S5 shows the DT models that delivered sensitivity at the 85%, 90%, and 95% levels, respectively. Then, we selected the 6 variables that were consistently present in all of these DT models. To evaluate the effectiveness of the proposed DT-based multivariate analysis, we further incorporated the LASSO^40^ and the mRMRe^41,42^ methods to extract another two 6-variable feature sets from the 18-variable feature set output by the first-stage feature selection process. Then, we proceeded to build the DT models, the LR models, and the DNN models based on these three 6-variable feature sets for performance evaluation.

**Table 1:** Results of the first-stage feature selection by logistic regression.

Item	Variables^a^	All patients N = 83,227	Estimate	Std. error	z value	Prob( >|z|)
0	(Intercept)		− 3.909283	0.037019	− 105.601	< 0.000
1	Age (mean ± SD)	51.08 ± 30.49	0.031406	0.000562	55.882	< 0.000
2	Heart disease	21,776 (26.16%)	0.448181	0.021741	20.615	< 0.000
3	Metastatic cancer	4264 (5.12%)	0.663727	0.037349	17.771	< 0.000
4	CVA	16,935 (20.35%)	0.361054	0.022895	15.77	< 0.000
5	Renal disease	6617 (7.95%)	0.429659	0.029803	14.417	< 0.000
6	Neurological disease	8443 (10.14%)	0.36544	0.028077	13.016	< 0.000
7	Diabetes	16,416 (19.72%)	0.280035	0.022435	12.482	< 0.000
8	Pulmonary disease	32,646 (39.23%)	− 0.243137	0.021165	− 11.488	< 0.000
9	Gender (male)	49,739 (59.76%)	0.227208	0.020821	10.912	< 0.000
10	Hypertension	30,663 (36.84%)	− 0.182299	0.02258	− 8.073	< 0.000
11	Tuberculosis	3083 (3.7%)	0.332174	0.042151	7.881	< 0.000
12	Cancer without metastasis	4113 (4.94%)	0.28759	0.038312	7.506	< 0.000
13	Leukemia and lymphoma	922 (1.11%)	0.565562	0.082878	6.824	< 0.000
14	Pregnancy/postpartum	334 (0.4%)	1.006209	0.167368	6.012	< 0.000
15	PVD	1689 (2.03%)	0.253768	0.05406	4.694	< 0.000
16	AIDS	63 (0.08%)	1.223306	0.306994	3.985	< 0.000
17	Severe liver disease	289 (0.35%)	0.496771	0.142543	3.485	0.000
18	Autoimmune disease	635 (0.76%)	0.262207	0.104528	2.508	0.012
19	Mental illness	1821 (2.19%)	0.100714	0.065314	1.542	0.123
20	Hypothyroidism	558 (0.67%)	0.137819	0.100471	1.372	0.170
21	Hyperthyroidism	542 (0.65%)	0.061082	0.112777	0.542	0.588

*CVA* cerebrovascular accident, *PVD* peripheral vascular disease, *AIDS* acquired immune deficiency syndrome.

### The development of prediction models

In this study, we followed the same rationale presented in our previous work^13^ to develop two types of machine learning-based prediction models, namely the DT^17–19^ and DNN models^15^. The performance of the DT models is of interest due to the explicit decision rules produced by the DT algorithm, which is a unique feature favored by clinicians. However, the algorithm for building a DT model is based on univariate analysis and does not incorporate any linear or non-linear transformation. As a result, the prediction performance of the DT models may not match the advanced prediction models when applied to those datasets in which different classes of samples are separated by non-linear boundaries. In this respect, with the advantage of non-linear transformations, the state-of-the-art DNN models generally can deliver superior prediction performance in comparison with other types of prediction models^45^. However, a DNN based model typically contains a large quantity of coefficients and therefore it is almost impossible for clinicians to figure out the logic embedded in the prediction process. In this study, we further investigated how the conventional LR models^43,46^ performed because logistic regression is widely used in medical and epidemiological research. Supplementary Table S4 summarizes the software packages and parameter settings employed to build the DT models and the main characteristics of the DNN models. With respect to the structure of the DNN models, we actually investigated the performance of more complicated networks and observed that the simple network structure shown in Supplementary Table S4 delivered the same level of performance in comparison with more complicated network structures. In this respect, we experimented with network dimensions of 8, 16, 24 and 32 and set the number of layers to 3 and 4.

### Model performance evaluation

To evaluate model performance, we employed 10-fold cross validation to evaluate the performance of our prediction models^44^. As shown in Supplementary Table S4, in order to generate the DT models with alternative performance characteristics, e.g. different levels of sensitivity, we set the prior and cp parameters to various values. For generating the LR models and the DNN models with alternative performance characteristics, we varied the cutoff values at the outputs in order to discretize the numerical outputs into binary states.

Model performance was evaluated based on several metrics, including accuracy, sensitivity, specificity, positive predicted value (PPV), negative predicted value (NPV), as well as three additional metrics designed to report the overall performance of the prediction models, namely, the F1 score^47^, the Matthews correlation coefficient (MCC)^47^, and the area under the receiver operating characteristics curve (AUC) (Supplementary Table S7). In the subsequent discussions regarding the performance delivered by various prediction models, we will focus on the F1 score, which is defined to be the the harmonic mean of the PPV and the sensitivity delivered by a prediction model and is a widely used performance metric in the machine learning research community. In recent years, scientists in the biomedical research communities have also started to incorporate the F1 score to report their performance data^48^.

## Results

To conduct a comprehensive performance analysis, we built different types of prediction models with alternative feature sets, Table 2 summaries the F1 scores delivered by these prediction models and the comprehensive performance data is shown in Supplementary Tables S7a–c. The alternative feature sets incorporated to build the prediction models included the three 6-variable feature sets identified by the proposed DT-based analysis, the mRMRe, and the LASSO, along with the 18-variable feature set identified by the logistic regression based analysis in the first stage of the feature selection process.

**Table 2:** The F-1 scores delivered by the alternative prediction models with different feature sets.

Model	Feature set	Sensitivity 85% Mean (95% confidence interval)	Sensitivity 90% Mean (95% confidence interval)	Sensitivity 95% Mean (95% confidence interval)
DT	6 features (by the proposed DT-based method)	0.446 (0.446–0.447)	0.436 (0.436–0.436)	0.410 (0.409–0.410)
	6 features (by mRMRe)	0.438 (0.438–0.438)	0.428 (0.428–0.428)	0.409 (0.409–0.409)
	6 features (by LASSO)	0.437 (0.437–0.437)	0.428 (0.427–0.428)	0.400 (0.400–0.400)
	18 features	0.442 (0.442–0.442)	0.437 (0.437–0.437)	0.413 (0.412–0.413)
LR	6 features (by the proposed DT-based method)	0.440 (0.440–0.441)	0.430 (0.430–0.430)	0.399 (0.399–0.400)
	6 features (by mRMRe)	0.435 (0.435–0.435)	0.423 (0.423–0.424)	0.398 (0.398–0.399)
	6 features (by LASSO)	0.433 (0.432–0.433)	0.421 (0.421–0.421)	0.397 (0.397–0.397)
	18 features	0.446 (0.446–0.446)	0.434 (0.434–0.434)	0.406 (0.406–0.406)
DNN	6 features (by the proposed DT-based method)	0.447 (0.447–0.447)	0.437 (0.437–0.437)	0.409 (0.408–0.409)
	6 features (by mRMRe)	0.438 (0.438–0.438)	0.431 (0.430–0.431)	0.410 (0.410–0.410)
	6 features (by LASSO)	0.437 (0.437–0.437)	0.428 (0.427–0.428)	0.400 (0.400–0.400)
	18 features	0.452 (0.451–0.452)	0.444 (0.443–0.444)	0.422 (0.421–0.422)

Please refer to Supplementary Table S6 for the definitions of the F1 score.

With respect to performance data shown in Table 2 and Supplementary Tables S7a–c, the first observation is that the DNN model built with 18 variables performed marginally superior to the other prediction models shown in Table 2. For example, under the column of 85% sensitivity, the F1 score of 0.452 delivered by the DNN model built with 18 variables is marginally higher than the other F1 scores delivered by the three DNN models built with the three different 6-variable feature sets, which were 0.447, 0.438, and 0.437, respectively. This observation implies that no significant information was lost when we employed only 6 variables. The second observation is that all these different types of prediction models built with alternative 6-variable feature sets basically delivered the same level of performance. For example, under the column with 85% sensitivity, the F1 scores delivered by different prediction models built with different 6-variable feature sets are all within the range from 0.433 to 0.447. Accordingly, in the following discussion, we will focus on the DT models built with 6 variables because the explicit prediction logic output by the DT algorithm was highly valuable with respect to clinical applications. The third observation is that the DT models built with the 6-variable feature set identified by the proposed DT-based method performed marginally superior to the DT models built with the 6-variables features sets identified by the mRMRe and the LASSO. For example, under the column with 85% sensitivity, the F1 scores delivered by the DT models built with the 6-variables features sets identified by the proposed DT-based method, the mRMRe, and the LASSO are 0.446, 0.438, and 0.437, respectively. While the discussions above focus on the F1 scores, Supplementary Fig. SF1 shows the receiver operating curves of alternative prediction models. Though we can observe marginal differences among the areas under the curve (AUCs) delivered by alternative prediction models, all receiver operating curves essentially overlap in the region above sensitivity 85%.

**Figure 3:**
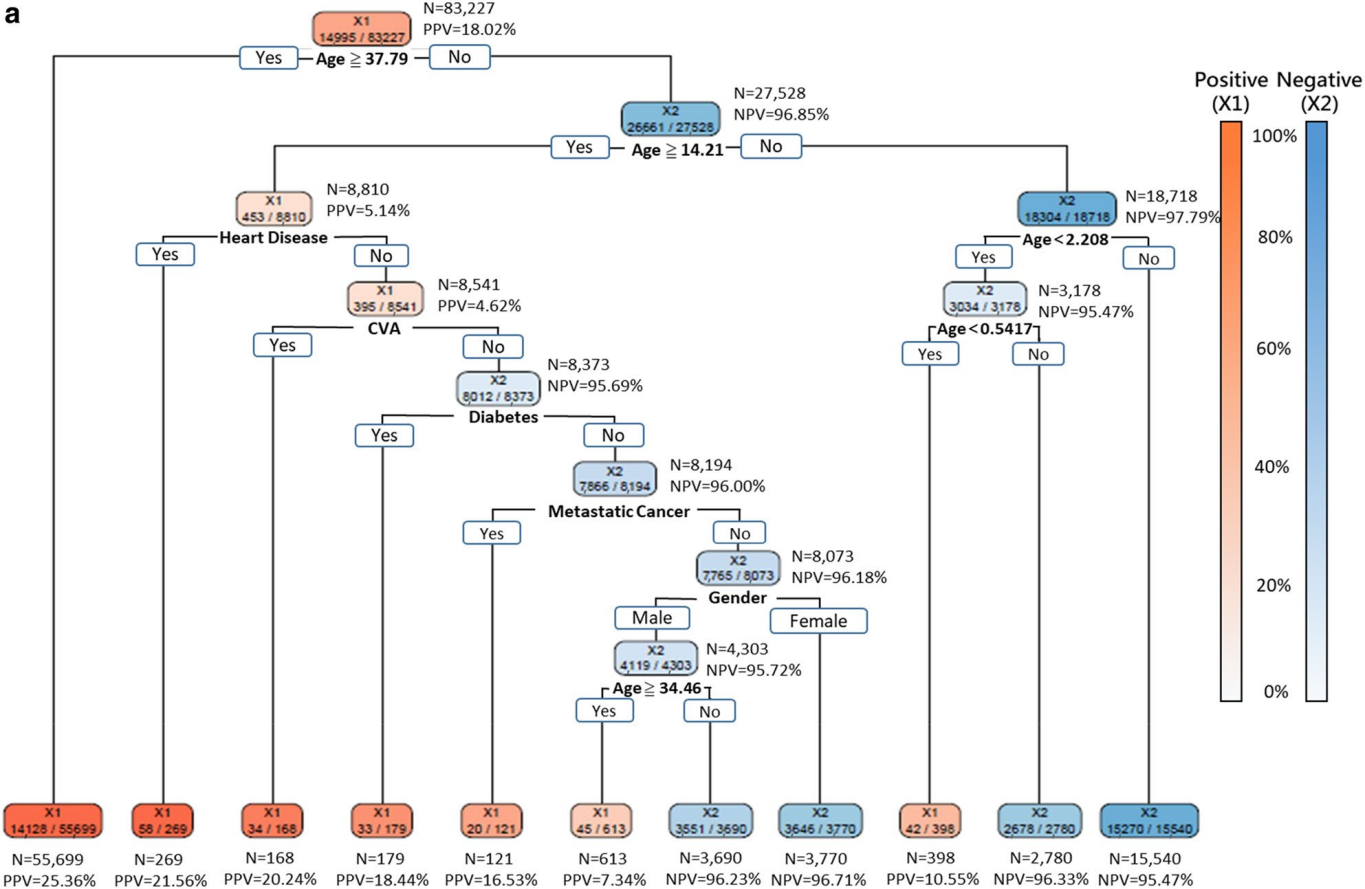

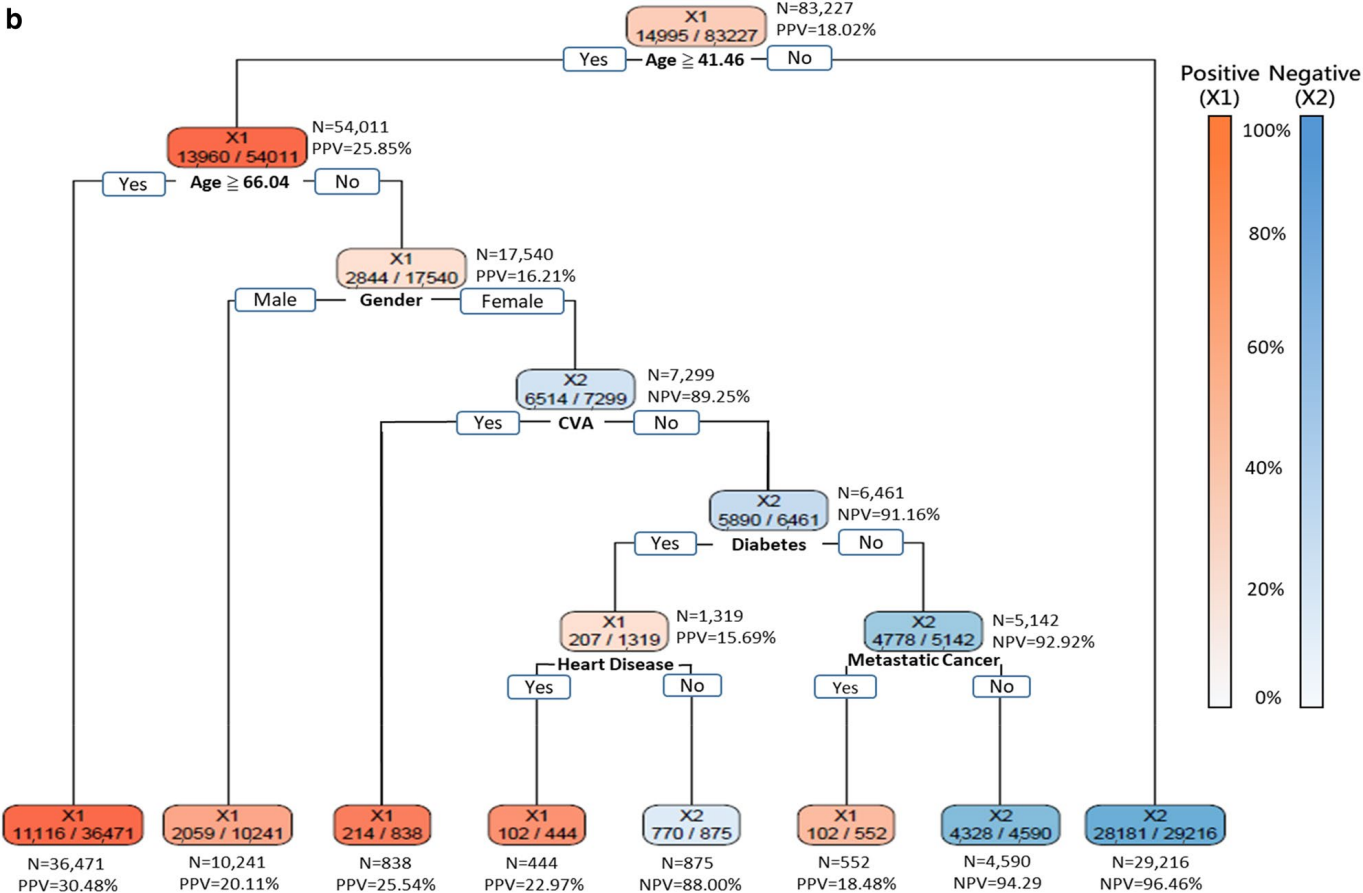

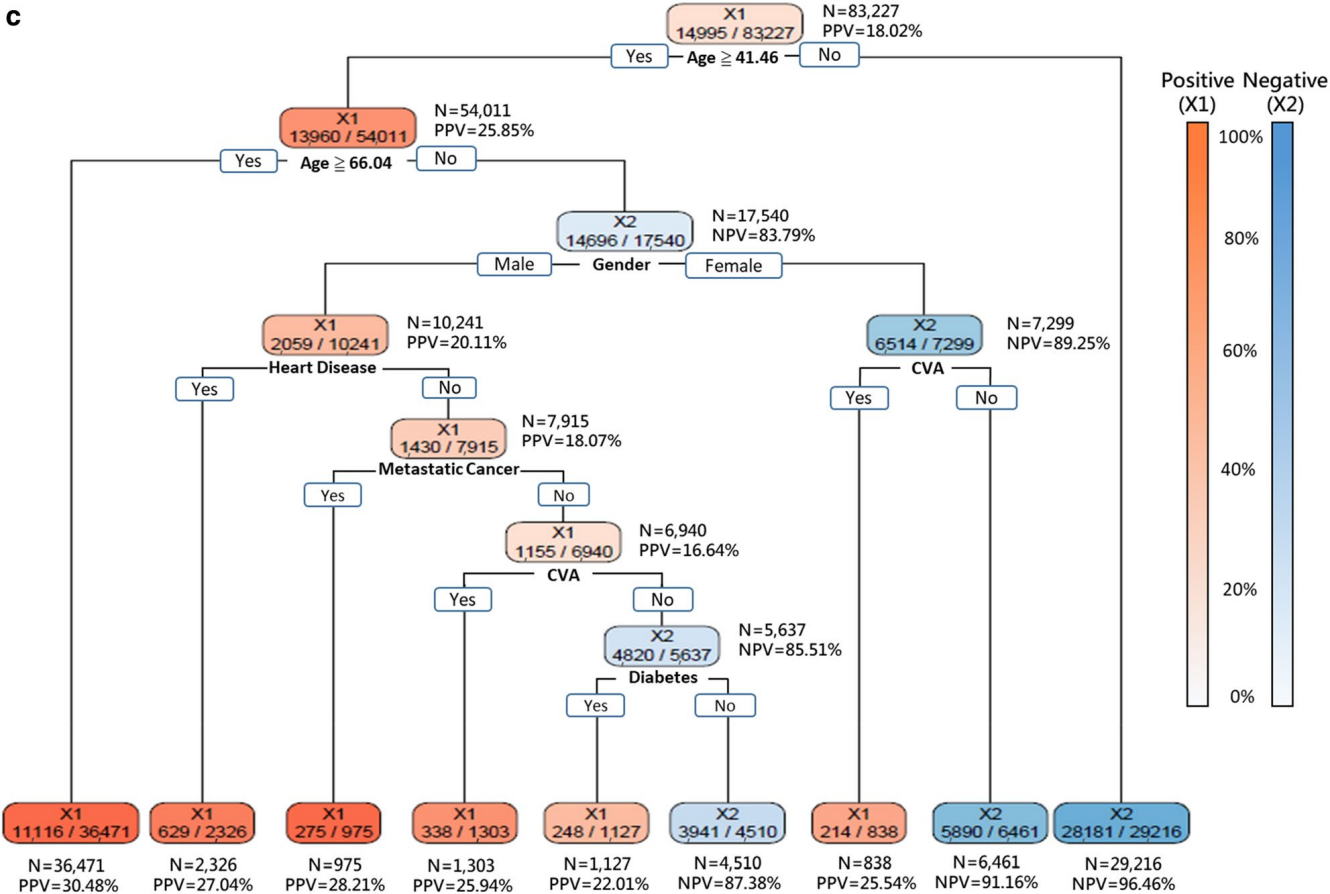
Decision trees generated with 6 features at three sensitivities (95%, 90%, 85%). (**a**) The decision tree built with the 6 features identified by the proposed DT-based method and delivering 95% sensitivity. (**b**) The decision tree built with the 6 features identified by the proposed DT-based method and delivering 90% sensitivity. (**c**) The decision tree built with the 6 features identified by the proposed DT-based method and delivering 85% sensitivity. *TP* true positive, *TN* true negative, *FP* false positive, *FN* false negative, *PPV* positive predictive value = TP/(TP + FP), *NPV* negative predictive value = TN/(TN + FN).

As decision-makers like to know how to allocate resources most appropriately under different scenarios, Fig. 3a–c shows the DT models that delivered 95%, 90%, and 85% sensitivities, respectively. Since age was placed at the top level of the tree structures in all these three models, it implied that age was the most crucial factor. The DT model with 95% sensitivity revealed that patients aged over 37.79 or under 0.54 years suffered high risk for severe ILI. Furthermore, the following two groups of patients also suffered high risk for severe ILI: (1) patients aged between 14.21 and 37.79 with heart disease, CVA, diabetes, metastatic cancer; and (2) male gender aged between 34.46 and 37.79 (Fig. 3a). The DT model with 90% sensitivity revealed that those patients older than 66.04 years-old suffered the highest risk of progression to severe illness. Furthermore, those female patients aged between 41.46 and 66.04 and with CVA, diabetes, heart disease, and metastatic cancer also suffered high risk for severe ILI (Fig. 3b). The DT model with 85% sensitivity identified the following three groups of patients that suffered high risk of severe ILI: (1) patients older than 66.04; (2) male patients aged between 41.46 and 66.04 with heart disease, metastatic cancer, CVA, and diabetes; and (3) female patients aged between 41.46 and 66.04 and with CVA (Fig. 3c). Overall, 31.0% (25,780/83,227), 41.7% (34,681/83,227) and 48.3% (40,187/83,227) of those hospitalized ILI patients were predicted to have low risk of progression to severe ILI by the three DT models with 95%, 90% and 85% sensitivity, respectively (Fig. 3).

Table 3 shows the relative risks and NPV delivered by the DT models with different levels of sensitivity. The relative risk compares the risk of progression to severe illness between the group of patients predicted by the DT model to be positive and the group of patients predicted to be negative. In field applications, the relative risk provides the public health administrators and the physicians with an instinctive understanding about how successfully the prediction model partitions the high-risk patients and the low-risk patients. As shown in Table 3, the relative risks delivered by the DT models with 95% sensitivity, 90% sensitivity, and 85% sensitivity were 10.15, 6.93, and 5.50, respectively, these values imply that the group of patients predicted by the DT models to be positive did in fact have significantly higher risk than the group of patients predicted to be negative. Table 3 also show that the NPVs of the DT models with different levels of sensitivity are all over 95%. The high NPVs imply that only very small percentages of the patients predicted to be negative were false negatives.

**Table 3:** The relative risks and negative predictive values delivered by the DT models with different levels of sensitivity.

Model sensitivity	TP	TN	FP	FN	Relative risk	NPV
85%	12,820	38,012	30,220	2175	5.50	94.59%
90%	13,593	33,279	34,953	1402	6.93	95.96%
95%	14,360	2,5145	43,087	635	10.15	97.54%

*TP* true positive, *TN* true negative, *FP* false positive, *FN* false negative, *NPV* negative predictive value.

Definitions: Relative risk = (TP/(TP + FP))/(FN/(TN + FN)); NPV = TN/(TN + FN).

Finally, as our cohort is imbalanced, containing 14,995 positive cases and 68,232 negative cases, we employed the random over-sampling examples (ROSE)^49^ package in R^50^ to address this issue. Supplementary Tables S7a–c show the results with the ROSE package incorporated. One obvious observation is that no significant difference exists between the data shown in Supplementary Tables S8a–c and those shown in Supplementary Tables S7a–c.

## Discussion

We have conducted a comprehensive analysis on how to exploit machine learning algorithms to stratify the risk of severe illness or death among hospitalized ILI patients. There were three major findings in this study. Firstly, the three different types of prediction models investigated in this study, namely the DNN models, the LR models, and the proposed DT based models, delivered comparable performance in predicting severe ILI after hospitalization. Secondly, the tree structures of the DT models explicitly illustrated how predictions were made and provide valuable guidelines for clinicians to develop effective strategies for risk stratification of ILI patients. Thirdly, the clinicians can employ the DT models with an appropriate sensitivity level to cope with the availability of medical resources and public health needs in different epidemic stages of an EID disaster.

With respect to the performance of the different types of prediction models, namely the DT models, the LR models, and the DNN models, our results may be confusing for some machine learning experts who strongly believe that the DNN models should prevail in most cases^17,45,51^. However, how the DNN model performs in comparison with different types of prediction models really depends on how different classes of subjects, e.g. positive vs. negative, are distributed in the dataset. If different classes of subjects can be partitioned by linear geometric objects defined by a very limited number of features, then different types of prediction models may deliver comparable performance. In other words, the DNN models may not prevail in this case, which was exactly what we observed in this study. In fact, we also observed a similar result from one of our recent studies on dengue^13^.

With the DT models being able to deliver performance comparable to the state-of-the-art DNN models, the explicit prediction rules presented in the DT structures provide valuable references for developing effective clinical strategies. All the studied DT models with different sensitivities identified age seniority as the most critical risk factor for severe ILI. This result is in conformity with clinical experience as advanced age, along with comorbid medical conditions such as diabetes^2,35^, cirrhosis^52^, malignant diseases^35,53^, etc., have been recognized as one of the crucial risk factors for severe ILI. Furthermore, the cutoffs employed by the DT models to partition age groups are in conformity with clinical insights. Nevertheless, these cutoffs along with the comorbidities identified in the DT structures provide clinicians with systematic clues regarding how to treat the patients most effectively when facing an EID.

There are two scenarios in which the DT models developed in this study can be exploited. The first scenario is that a public health administrator may want to develop an effective vaccination policy. In this scenario, the decision rules output by the DT models can provide the health policymaker with a set of guidelines for prioritizing the groups of people with a high risk of disease progression to receive the vaccine. In this respect, as shown in Table 3, the relative risks delivered by the DT models with different levels of sensitivity were all over 5, which implies that the group of patients predicted to be positive suffered a significantly high risk of progression than the group of patients predicted to be negative. Depending on the coverage of the high-risk population to be achieved, the public health administrator can decide which DT model should be employed. For example, when the vaccine is just successfully developed, the quantity of the vaccine available may be limited. In this case, the public health administrators can adopt the decision rules provided by the DT model with a lower sensitivity, e.g. 85%. Once the production of the vaccine runs smoothly and there is an abundance of vaccine, the decision rules provided by the DT model with 95% sensitivity can be exploited to achieve herd immunity. In addition to the application described above, the decision rules output by the DT models can provide the general public with valuable health guidelines. These decision rules can remind those people with high risk to watch their health conditions closely and seek medical help once they suffer from mild symptoms.

**Figure 4:**
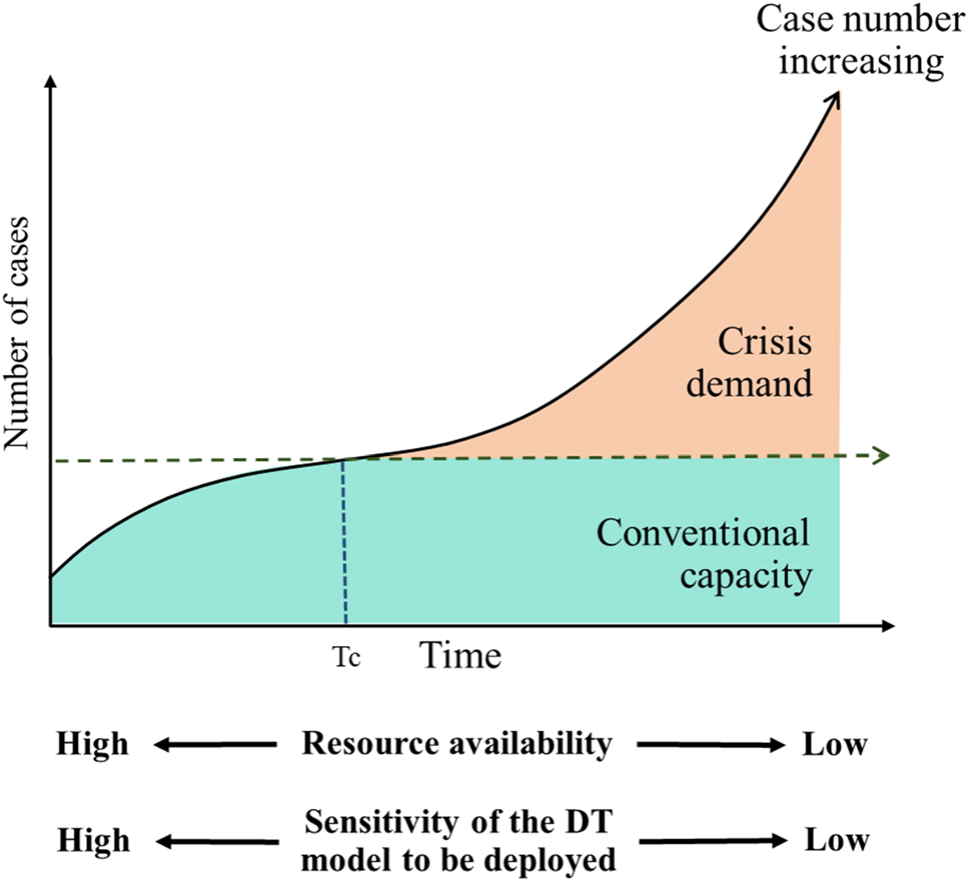
The curve that shows how the number of cases increases as the EID disaster progresses^61^. Beyond time point of a crisis (T_c_), the medical facilities start to operate under a crisis mode. In the early stage of an EID disaster, when the healthcare capacity is adequate, the DT models with high sensitivity levels should be employed to identify patients with risk of infection progression for them to be hospitalized and receive the best treatment^9,54^. In the later stages of an EID disaster, the available medical resources may be exhausted due to a tremendous increase of patients. In this scenario, the DT models with a lower sensitivity should be employed to recommend only those patients with a high risk of progressing to severe infection or death for hospitalization and thereby avoid collapse of medical facilities.

Another scenario in which the prediction models developed in this study could be incorporated is to optimize resource management at healthcare facilities once an EID disaster emerges. The DT models with different levels of sensitivity can be employed in different stages of an EID disaster (Fig. 4). In the early stage of an EID disaster, when the healthcare capacities are not overloaded, the DT model with 95% sensitivity should be employed to identify patients with risk of disease progression so that they can be hospitalized and receive the best possible treatment^9,54^ to minimize fatalities. As shown in Table 3, the DT model with 95% sensitivity could discharge 30.9% (25,780/83,227) of the admitted ILI patients from medical facilities with only 0.8% (635/83,227) patients were mistakenly discharged. As the development of the EID disaster progresses, the tremendous increase of the patient number and the surging demands for medical resources may rapidly exceed the capacities of medical facilities. In the recent COVID-19 pandemic, almost all countries with community outbreaks experienced unprecedented mortality due to the collapse of the healthcare systems. In this event, clinicians may be forced to triage patients without laboratory tests, which could become scarce during a pandemic, in order to discharge patients without potential risk for subsequent deterioration^55^. Accordingly, the DT model with 85% sensitivity can be employed, which predicted 48.3% (40,187/83,227) of the admitted ILI patients to be without risk of progression and could be discharged to relieve the overload at medical facilities. The high NPV value delivered by the DT model with 85% sensitivity, which was 94.6% as shown in Table 3, suggests that only a small percentage of patients would be mistakenly discharged.

There are several limitations in the current study. Firstly, the diagnosis of ILI was based on ICD-9-CM codes without laboratory confirmation of influenza. Nevertheless, ILI-related clinical syndromes may be the best surrogate diagnostic category representative of patients with community-onset respiratory infections that may progress towards severe illness and death^33,35^. Secondly, our dataset based on nation-wide insurance reimbursement data (claims data) does not include laboratory data, and other potential confounding factors that may influence the prognosis of respiratory infections, including obesity^56,57^, smoking^58^, geographic distributions^59^, and social economic conditions^60^, which were not available in the NHIRD database. However, our model based on demographic data and comorbidities is useful in preventive measurements, such as public education and vaccination policy. Furthermore, physicians under shortage of resources during the pandemic have to use fewer laboratory test results to identify the population at risk. Thirdly, we did not investigate the performance of other advanced machine learning algorithms such as the support vector machine, random forests, Bayesian networks, etc. Nevertheless, it is generally observed that the DNN based prediction models can deliver comparable or ever superior performance when compared with other advanced machine learning algorithms. Fourthly, as our experimental data was extracted from a single national insurance reimbursement database, readers should be cautious to generalize our findings before further validation studies are conducted.

In conclusion, our results showed that the DT-based prediction models delivered performance comparable to the DNN models in predicting ILI severity. The explicit prediction logic shown in the DT structures may be exploited to facilitate the decision-making process executed by clinicians. Furthermore, the DT models with alternative sensitivity levels can be exploited in different stages of an EID disaster to optimize medical resource allocation, which is crucial in the response to a large-scale epidemic of emerging infectious disease.

## Supplementary Information

Supplementary Information.
