# Supplementary material for: Machine learning for emerging infectious disease field responses
Source: Sci Rep. 2022 Jan 10;12:328. doi: 10.1038/s41598-021-03687-w (PMC8748708; doi:10.1038/s41598-021-03687-w)
Supplement: Supplementary file 1 — Supplementary Information. [file 41598_2021_3687_MOESM1_ESM.pdf]

Supplementary material

Table S1. The ICD-9-CM (International Classification of Disease, 9th Revision, Clinical Modification) codes of Influenza-like illness (ILI)

ICD-9 code	Description
**079.99**	Unspecified virus infection in condition classified elsewhere and of unspecified site
**307.81**	Tension headache
**372.3**	Other and unspecified conjunctivitis
**382.9**	Unspecified otitis media
**460**	Acute nasopharyngitis [common cold]
**461.1**	Acute sinusitis frontal
**461.9**	Acute sinusitis, unspecified
**462**	Acute pharyngitis
**463**	Acute tonsillitis
**464**	Acute laryngitis and tracheitis
**464.4**	Croup
**465**	Acute upper respiratory infections of multiple or unspecified site
**465.8**	Acute upper respiratory infections of other multiple sites
**465.9**	Acute upper respiratory infections of unspecified site
**466**	Acute bronchitis and bronchiolitis
**466.0**	Acute bronchitis
**466.19**	Acute bronchiolitis due to other infectious organisms
**472**	Chronic pharyngitis and nasopharyngitis
**477.9**	Allergic rhinitis cause unspecified
**482.31**	Pneumonia due to Streptococcus, Group A
**482.32**	Pneumonia due to Streptococcus, Group B
**482.39**	Pneumonia due to other Streptococcus
**482.4**	Pneumonia due to Staphylococcus
**482.41**	Pneumonia due to Staphylococcus aureus
**482.49**	Pneumonia due to other Staphylococcus
**482.81**	Pneumonia due to Anaerobes
**482.82**	Pneumonia due to Escherichia coli (E. coli)
**482.83**	Pneumonia due to other gram-negative bacteria
**482.84**	Legionnaires' disease
**482.89**	Pneumonia due to other specified bacteria
**483**	Pneumonia due to other specified organism
**483.1**	Pneumonia due to Chlamydia
**484.1**	Pneumonia in cytomegalic inclusion disease
**484.3**	Pneumonia in whooping cough

ICD-9 code	Description
**484.6**	Pneumonia in aspergillosis
**484.7**	Pneumonia in other systemic mycoses
**486**	Pneumonia, organism unspecified
**487**	Influenza
**487.0**	Influenza with pneumonia
**487.1**	Influenza with other respiratory manifestations
**487.8**	Influenza with other manifestations
**490**	Bronchitis, not specified as acute or chronic
**496**	Chronic airways obstruction, not elsewhere classified
**780.6**	Fever
**780.79**	Other malaise and fatigue
**782.5**	Cyanosis
**784**	Symptoms involving head and neck
**785.6**	Enlargement of lymph nodes
**786.07**	Wheezing
**786.09**	Other dyspnea and respiratory abnormalities
**786.2**	Cough
**786.5**	Chest pain
**787.02**	Nausea alone
**787.03**	Vomiting alone

Table S2. The ICD-9-CM (International Classification of Disease, 9th Revision, Clinical Modification) codes for the 19 comorbidities incorporated in this study.

No.	Comorbidity*	ICD-9-CM codes	Catastrophic illness codes #
1	Heart disease	394.x, 395.x, 396.x, 398.91, 402.0, 402.11, 402.91, 404.01, 404.11, 404.13, 404.91, 404.93, 410.x, 412, 411.x, 413.x, 414.x, 424.0, 424.1, 424.2, 424.3, 427.0, 427.2, 427.3, 427.31,427.32, 427.4x, 427.5, 427.6x, 427.8x, 427.9x, 428.x	
2	PVD	440.2x, 440.3x, 443.x, 444.2x, 447.1, 785.4, V43.4	
3	Hypertension	401.x, 402.x, 403.x, 404.x, 405.x, 437.2	
4	CVA	342.x, 430, 431, 432.x, 433.x, 434.x, 435.x, 436, 437.x, 438.x, Procedure 38.48	
5	Neurological disease	Dementia: 290.x, 331.0, 331.2; Epilepsy: 345.x	
6	Pulmonary disease	COPD: 490, 491.x, 492.x, 494.x, 496, 500, 501, 502, 503, 504, 505, 506.4; Asthma: 493.x	
7	Autoimmune disease	340.x, 694.4, 694.5, 696.0, 696.1, 696.8, 710.x, 714.x, 720.x, 724.x	05 (Autoimmune disease); 21 (Multiple sclerosis)
8	Severe liver disease	070.22, 070.23, 070.32, 070.33, 070.54, 456.0, 456.1, 456.20, 456.21, 571.x, 573.x, 572.2-572.8	25 (Liver Cirrhosis)
9	Diabetes	250.0x, 250.1x, 250.2x, 250.3x, 250.8x, 250.9x, 250.4x, 250.5x, 250.6x, 250.7x	
10	Hyperthyroidism	242.x	
11	Hypothyroidism	243.x, 244.x	
12	Renal disease	403.11, 403.91, 404.12, 404.13, 404.92, 404.93, 582.x, 583.x, 585, 586, 588.x	
13	Cancer without metastasis	140.x-195.x<br>✻Patients without code 196.x-199.x and 200.xx-208.xx	01 (Cancer)
14	Metastatic cancer	196.x-199.x	01 (Cancer)
15	Leukemia and lymphoma	200.xx-208.xx<br>✻Patients with code 200.xx-208.xx, even though they have code 140.x-195.x and 196.x-199.x.	01 (Cancer)
16	AIDS	042	
17	Tuberculosis	010.xx-018.xx	
18	Mental illness	295.x-299.x	06 (Chronic mental illness)
19	Pregnancy/ postpartum	630-679<br>Hospital Order Code: 57114C, 57115C, 81004C, 81005B, 81005C, 81010C, 81017C, 81024C, 81025C, 81026C, 81028C, 81029C, 81034C, 97001A, 97001K, 97002A, 97003B, 97004C, 97005D, 97006K, 97007A, 97008B, 97009C, 97014C, 97931K, 97932A, 97933B, 97934C, 98001K, 98002A, 98003B, 98004C, P3503C, P3504C	

*Abbreviations: AIDS (acquired immune deficiency syndrome); COPD (chronic obstructive pulmonary disease); CVA (cerebrovascular accident); PVD (peripheral vascular disease).

#Patients with these comorbidities were confirmed with records on catastrophic illness card through National Health Insurance in Taiwan, and the specific code number represented its own category of catastrophic Illness and subsidies.

Table S3. Demographic analyses of the cohort

Classification	Variables	All patients N=83,227	Severe or Fatal cases# N=14,995 (18.02%)	Fatal cases N=5,282 (6.35%)
Age(mean ± SD)		51.08 ± 30.49	71.58 ± 18.70	75.48 ± 14.62
Gender				
	Male	49,739	9,827 (19.76%)	3,544 (7.13%)
	Female	33,488	5,168 (15.43%)	1,738 (5.19%)
Comorbidity*				
	1 Heart Disease	21,776	7,028 (32.27%)	2,400 (11.02%)
	2 PVD	1,689	607 (35.94%)	225 (13.32%)
	3 Hypertension	30,663	8,388 (27.36%)	2,919 (9.52%)
	4 CVA	16,935	5,628 (33.23%)	1,971 (11.64%)
	5 Neurological Disease	8,443	2,897 (34.31%)	1,023 (12.12%)
	6 Pulmonary Disease	32,646	7,363 (22.55%)	2,521 (7.72%)
	7 Autoimmune Disease	635	126 (19.84%)	49 (7.72%)
	8 Severe Liver Disease	289	69 (23.88%)	27 (9.34%)
	9 Diabetes	16,416	4,982 (30.35%)	1,733 (10.56%)
	10 Hyperthyroidism	542	113 (20.85%)	41 (7.56%)
	11 Hypothyroidism	558	158 (28.32%)	73 (13.08%)
	12 Renal Disease	6,617	2,370 (35.82%)	927 (14.01%)
	13 Cancer without Metastasis	4,113	1,131 (27.5%)	529 (12.86%)
	14 Metastatic Cancer	4,264	1,245 (29.2%)	850 (19.93%)
	15 Leukemia and Lymphoma	922	223 (24.19%)	137 (14.86%)
	16 AIDS	63	15 (23.81%)	9 (14.29%)
	17 Tuberculosis	3,083	974 (31.59%)	408 (13.23%)
	18 Mental Illness	1,821	336 (18.45%)	125 (6.86%)
	19 Pregnancy/ Postpartum	334	42 (12.57%)	0 (0%)

*Abbreviations: AIDS (acquired immune deficiency syndrome); CVA (cerebrovascular accident); PVD (peripheral vascular disease).

Severe cases: Hospitalized patients requiring critical cares such as intubation, ventilator support, extracorporeal membrane oxygenation treatment, admission to an intensive care unit during the hospitalization period.

Table S4. Parameter settings for the DT and DNN models

Model	Package	Parameter Setting
**DT**	**R Package rpart**	split = Information
		minbuck = 100
		xval = 10
		prior = 0.35 ~ 0.85 with 0.01 resolution
		cp = 0.001~0.008 with0.001 resolution
**Multilayer DNN**	**Python Keras**	Input neurons: 6 or 18 corresponding to 6 or 18 features.
		hidden layers: 3 or 4
		hidden neurons: 8,16, 24, 32.
		1 output neuron

Table S5. Characteristics of the three DT models incorporated in the proposed DT-based multivariate analysis

	Model with 85.50% sensitivity	Model with 90.82% sensitivity	Model with 95.95% sensitivity
Parameter values	prior=0.51.<br>cp=0.0040	prior=0.60<br>cp=0.0007	prior=0.81<br>cp=0.0008
Features	1. Age<br>2. Gender<br>3. Heart Disease<br>4. Metastatic Cancer<br>5. CVA<br>6. Diabetes	1.Age<br>2.Gender<br>3.Heart Disease<br>4.Metastatic Cancer<br>5.CVA<br>6.Diabetes<br>7.Cancer without Metastasis<br>8.Renal Disease<br>9.Neurological Disease	1.Age<br>2.Gender<br>3.CVA<br>4.Diabetes<br>5.Metastatic Cancer<br>6.Heart Disease<br>7.Autoimmune Disease<br>8.Neurological Disease<br>9.Pregnancy/ Postpartum<br>10. Pulmonary Disease

Note:

1. The following six features were present in all three DT models with different levels of sensitivity: age, gender, heart disease, metastatic cancer, cerebrovascular accident (CVA), and diabetes.

Table S6. Definitions of performance metrics

metric	Definition
TP	True positive
TN	True negative
FP	False positive
FN	False negative
Accuracy	(TP+TN)/(TP+FP+TN+FN)
Sensitivity	TP/(TP+FN)
Specificity	TN/(TN+FP)
PPV	TP/(TP+FP)
NPV	TN/(TN+FN)
F1 score	2TP/(2TP+FP+FN)
MCC	Matthews correlation coefficient

Abbreviations: PPV (positive predictive value); NPV (negative predictive value).

Table S7(a). Detailed performance data of the DT prediction models

Model	Feature set	Performance metric	Model with 85% sensitivity	Model with 90% sensitivity	Model with 95% sensitivity
**DT**	**6 features (by the proposed DT- based method)**	Accuracy	61.352% (61.291% - 61.413%)	57.780% (57.717% - 57.844%)	49.041% (48.826% - 49.257%)
		PPV	29.982% (29.955% - 30.009%)	28.605% (28.584% - 28.627%)	25.510% (25.433% - 25.586%)
		Sensitivity	85.751% (85.665% - 85.838%)	89.802% (89.714% - 89.889%)	95.212% (95.166% - 95.258%)
		Specificity	55.990% (55.900% - 56.080%)	50.743% (50.648% - 50.838%)	38.895% (38.623% - 39.167%)
		F1	0.444 (0.444 - 0.445)	0.434 (0.434 - 0.434)	0.402 (0.401 - 0.403)
		MCC	0.321 (0.321 - 0.321)	0.314 (0.314 - 0.315)	0.279 (0.278 - 0.281)
		**AUC**	**0.741 (0.740 - 0.742)**	**0.728 (0.728 - 0.728)**	**0.715 (0.712 - 0.718)**
	**6 features (by mRMRe)**	Accuracy	60.278% (60.170% - 60.385%)	56.655% (56.516% - 56.795%)	50.130% (50.058% - 50.201%)
		PPV	29.289% (29.250% - 29.329%)	28.084% (28.040% - 28.128%)	25.914% (25.889% - 25.938%)
		Sensitivity	85.181% (85.026% - 85.335%)	90.065% (89.882% - 90.248%)	95.105% (95.069% - 95.140%)
		Specificity	54.805% (54.641% - 54.969%)	49.313% (49.104% - 49.522%)	40.246% (40.152% - 40.340%)
		F1	0.436 (0.436 - 0.436)	0.428 (0.428 - 0.428)	0.407 (0.407 - 0.408)
		MCC	0.308 (0.307 - 0.308)	0.306 (0.306 - 0.307)	0.287 (0.287 - 0.287)
		**AUC**	**0.726 (0.726 - 0.726)**	**0.724 (0.724 - 0.724)**	**0.726 (0.725 - 0.727)**
	**6 features (by LASSO)**	Accuracy	60.287% (60.203% - 60.370%)	55.878% (55.800% - 55.957%)	48.204% (48.177% - 48.231%)
		PPV	29.300% (29.274% - 29.327%)	27.791% (27.760% - 27.821%)	25.249% (25.241% - 25.257%)
		Sensitivity	85.227% (85.068% - 85.386%)	90.649% (90.591% - 90.707%)	95.630% (95.610% - 95.651%)
		Specificity	54.806% (54.671% - 54.941%)	48.237% (48.129% - 48.344%)	37.782% (37.745% - 37.818%)
		F1	0.436 (0.436 - 0.436)	0.425 (0.425 - 0.426)	0.400 (0.399 - 0.400)
		MCC	0.308 (0.308 - 0.308)	0.304 (0.303 - 0.304)	0.276 (0.276 - 0.276)
		**AUC**	**0.725 (0.724 - 0.725)**	**0.728 (0.728 - 0.728)**	**0.670 (0.670 - 0.671)**
	**18-features**	Accuracy	61.200% (61.005% - 61.396%)	57.971% (57.903% - 58.040%)	49.887% (49.691% - 50.082%)
		PPV	29.762% (29.683% - 29.840%)	28.777% (28.753% - 28.802%)	25.912% (25.841% - 25.983%)
		Sensitivity	84.800% (84.541% - 85.059%)	90.357% (90.270% - 90.444%)	95.808% (95.767% - 95.848%)
		Specificity	56.014% (55.720% - 56.308%)	50.854% (50.753% - 50.955%)	39.795% (39.549% - 40.041%)
		F1	0.441 (0.440 - 0.441)	0.437 (0.436 - 0.437)	0.408 (0.407 - 0.409)
		MCC	0.314 (0.313 - 0.314)	0.320 (0.319 - 0.320)	0.290 (0.289 - 0.291)
		**AUC**	**0.723 (0.723 - 0.723)**	**0.730 (0.730 - 0.730)**	**0.718 (0.715 - 0.720)**

Note: Please refer to supplementary Table S6 for the definitions of performance metrics. Abbreviation: AUC: receiver operating characteristic curve area under the curve.

Table S7(b). Detailed performance data of the LR prediction models.

Model	Feature set	Performance metric	Model with 85% sensitivity	Model with 90% sensitivity	Model with 95% sensitivity
LR	**6 features (by the proposed DT- based method)**	Accuracy	61.076% (61.052% - 61.100%)	56.970% (56.945% - 56.995%)	48.619% (48.543% - 48.695%)
		PPV	29.716% (29.701% - 29.730%)	28.228% (28.216% - 28.240%)	25.321% (25.293% - 25.350%)
		Sensitivity	84.995% (84.994% - 84.996%)	89.999% (89.997% - 90.001%)	94.997% (94.996% - 94.999%)
		Specificity	55.820% (55.790% - 55.849%)	49.712% (49.682% - 49.742%)	38.427% (38.335% - 38.520%)
		F1	0.440 (0.440 - 0.441)	0.430 (0.430 - 0.430)	0.400 (0.399 - 0.400)
		MCC	0.314 (0.314 - 0.314)	0.309 (0.308 - 0.309)	0.274 (0.274 - 0.275)
		**AUC**	**0.756 (0.755 - 0.756)**	**0.756 (0.755 - 0.756)**	**0.756 (0.755 - 0.756)**
	**6 features (by mRMRe)**	Accuracy	60.188% (60.166% - 60.210%)	55.851% (55.822% - 55.880%)	48.344% (48.299% - 48.389%)
		PPV	29.212% (29.200% - 29.224%)	27.689% (27.676% - 27.703%)	25.218% (25.201% - 25.235%)
		Sensitivity	84.994% (84.990% - 84.998%)	90.004% (90.001% - 90.007%)	94.997% (94.995% - 94.999%)
		Specificity	54.736% (54.710% - 54.763%)	48.345% (48.309% - 48.381%)	38.091% (38.036% - 38.146%)
		F1	0.435 (0.435 - 0.435)	0.423 (0.423 - 0.424)	0.399 (0.398 - 0.399)
		MCC	0.306 (0.306 - 0.306)	0.299 (0.299 - 0.299)	0.272 (0.272 - 0.273)
		**AUC**	**0.751 (0.751 - 0.751)**	**0.751 (0.751 - 0.751)**	**0.751 (0.751 - 0.751)**
	**6 features (by LASSO)**	Accuracy	59.842% (59.817% - 59.867%)	55.450% (55.407% - 55.494%)	47.981% (47.923% - 48.039%)
		PPV	29.021% (29.007% - 29.034%)	27.501% (27.481% - 27.521%)	25.084% (25.063% - 25.105%)
		Sensitivity	84.995% (84.993% - 84.998%)	90.001% (89.999% - 90.004%)	94.997% (94.995% - 94.999%)
		Specificity	54.315% (54.284% - 54.345%)	47.857% (47.805% - 47.910%)	37.649% (37.578% - 37.719%)
		F1	0.433 (0.433 - 0.433)	0.421 (0.421 - 0.422)	0.397 (0.397 - 0.397)
		MCC	0.303 (0.302 - 0.303)	0.296 (0.295 - 0.296)	0.269 (0.269 - 0.270)
		**AUC**	**0.755 (0.755 - 0.755)**	**0.755 (0.755 - 0.755)**	**0.755 (0.755 - 0.755)**
	**18-features**	Accuracy	62.038% (62.008% - 62.067%)	57.642% (57.608% - 57.675%)	49.861% (49.796% - 49.925%)
		PPV	30.280% (30.263% - 30.298%)	28.562% (28.545% - 28.579%)	25.795% (25.770% - 25.820%)
		Sensitivity	84.995% (84.995% - 84.995%)	89.997% (89.996% - 89.998%)	94.998% (94.997% - 94.999%)
		Specificity	56.992% (56.957% - 57.028%)	50.531% (50.490% - 50.572%)	39.941% (39.862% - 40.020%)
		F1	0.447 (0.446 - 0.447)	0.434 (0.433 - 0.434)	0.406 (0.405 - 0.406)
		MCC	0.323 (0.322 - 0.323)	0.314 (0.314 - 0.315)	0.284 (0.284 - 0.285)
		**AUC**	**0.767 (0.767 - 0.767)**	**0.767 (0.767 - 0.767)**	**0.767 (0.767 - 0.767)**

Note: Please refer to supplementary Table S6 for the definitions of performance metrics. Abbreviation: AUC: receiver operating characteristic curve area under the curve.

Table S7(c). Detailed performance data of the DNN prediction models.

Model	Feature set	Performance metric	Model with 85% sensitivity	Model with 90% sensitivity	Model with 95% sensitivity
**DNN**	**6 features (by the proposed DT- based method)**	Accuracy	62.090% (62.065% - 62.115%)	58.168% (58.138% - 58.197%)	50.449% (50.376% - 50.523%)
		PPV	30.312% (30.297% - 30.327%)	28.829% (28.814% - 28.844%)	26.026% (25.997% - 26.055%)
		Sensitivity	84.996% (84.994% - 84.999%)	90.000% (89.998% - 90.003%)	94.998% (94.996% - 95.000%)
		Specificity	57.055% (57.025% - 57.086%)	51.172% (51.135% - 51.208%)	40.659% (40.570% - 40.749%)
		F1	0.447 (0.447 - 0.447)	0.437 (0.437 - 0.437)	0.409 (0.408 - 0.409)
		MCC	0.323 (0.323 - 0.323)	0.319 (0.319 - 0.319)	0.289 (0.288 - 0.289)
		**AUC**	**0.759 (0.759 - 0.759)**	**0.759 (0.759 - 0.759)**	**0.759 (0.759 - 0.759)**
	**6 features (by mRMRe)**	Accuracy	60.755% (60.731% - 60.779%)	57.117% (57.088% - 57.147%)	50.733% (50.678% - 50.788%)
		PPV	29.532% (29.518% - 29.546%)	28.302% (28.287% - 28.316%)	26.139% (26.117% - 26.160%)
		Sensitivity	84.998% (84.995% - 85.001%)	90.006% (90.003% - 90.009%)	94.999% (94.997% - 95.002%)
		Specificity	55.427% (55.398% - 55.457%)	49.890% (49.854% - 49.926%)	41.005% (40.938% - 41.072%)
		F1	0.438 (0.438 - 0.438)	0.431 (0.430 - 0.431)	0.410 (0.410 - 0.410)
		MCC	0.311 (0.311 - 0.311)	0.310 (0.310 - 0.310)	0.291 (0.291 - 0.291)
		**AUC**	**0.757 (0.757 - 0.757)**	**0.757 (0.757 - 0.757)**	**0.757 (0.757 - 0.757)**
	**6 features (by LASSO)**	Accuracy	60.866% (60.827% - 60.906%)	56.950% (56.913% - 56.988%)	49.935% (49.874% - 49.996%)
		PPV	29.595% (29.572% - 29.618%)	28.219% (28.201% - 28.237%)	25.824% (25.800% - 25.847%)
		Sensitivity	84.995% (84.992% - 84.999%)	90.005% (90.002% - 90.007%)	94.999% (94.996% - 95.003%)
		Specificity	55.563% (55.515% - 55.612%)	49.686% (49.641% - 49.732%)	40.031% (39.956% - 40.105%)
		F1	0.439 (0.439 - 0.439)	0.430 (0.429 - 0.430)	0.406 (0.406 - 0.406)
		MCC	0.312 (0.312 - 0.312)	0.309 (0.308 - 0.309)	0.285 (0.284 - 0.285)
		**AUC**	**0.762 (0.762 - 0.762)**	**0.762 (0.762 - 0.762)**	**0.762 (0.762 - 0.762)**
	**18-features**	Accuracy	62.800% (62.741% - 62.860%)	59.349% (59.297% - 59.400%)	53.034% (52.988% - 53.081%)
		PPV	30.744% (30.708% - 30.781%)	29.447% (29.420% - 29.475%)	27.091% (27.071% - 27.110%)
		Sensitivity	84.995% (84.995% - 84.995%)	89.997% (89.996% - 89.998%)	94.999% (94.998% - 95.000%)
		Specificity	57.923% (57.850% - 57.995%)	52.613% (52.551% - 52.676%)	43.812% (43.756% - 43.868%)
		F1	0.452 (0.451 - 0.452)	0.444 (0.443 - 0.444)	0.422 (0.421 - 0.422)
		MCC	0.330 (0.329 - 0.330)	0.329 (0.329 - 0.330)	0.309 (0.309 - 0.310)
		**AUC**	**0.772 (0.772 - 0.772)**	**0.772 (0.772 - 0.772)**	**0.772 (0.772 - 0.772)**

Note: Please refer to supplementary Table S6 for the definitions of performance metrics. Abbreviation: AUC: receiver operating characteristic curve area under the curve.

Table S8(a). Detailed performance data of the DT prediction models with the ROSE package incorporated

Model	Feature set	Performance metric	Model with 85% sensitivity	Model with 90% sensitivity	Model with 95% sensitivity
**DT (ROSE)**	**6 features (by the proposed DT- based method)**	Accuracy	61.664% (61.593% - 61.736%)	57.471% (57.431% - 57.510%)	49.678% (49.548% - 49.808%)
		PPV	30.071% (30.040% - 30.101%)	28.504% (28.491% - 28.518%)	25.715% (25.669% - 25.762%)
		Sensitivity	85.078% (84.992% - 85.164%)	90.205% (90.158% - 90.252%)	94.930% (94.899% - 94.962%)
		Specificity	56.519% (56.413% - 56.625%)	50.277% (50.218% - 50.335%)	39.733% (39.567% - 39.898%)
		F1	0.444 (0.444 - 0.445)	0.433 (0.433 - 0.433)	0.405 (0.404 - 0.405)
		MCC	0.320 (0.320 - 0.320)	0.314 (0.314 - 0.314)	0.282 (0.281 - 0.283)
		**AUC**	**0.737 (0.736 - 0.737)**	**0.729 (0.729 - 0.729)**	**0.711 (0.709 - 0.713)**
	**6 features (by mRMRe)**	Accuracy	60.252% (60.150% - 60.353%)	56.679% (56.568% - 56.790%)	49.125% (48.899% - 49.350%)
		PPV	29.225% (29.196% - 29.255%)	28.092% (28.062% - 28.121%)	25.553% (25.477% - 25.628%)
		Sensitivity	84.836% (84.638% - 85.033%)	90.037% (89.860% - 90.214%)	95.298% (95.223% - 95.373%)
		Specificity	54.849% (54.682% - 55.016%)	49.349% (49.174% - 49.523%)	38.977% (38.686% - 39.269%)
		F1	0.435 (0.435 - 0.435)	0.428 (0.428 - 0.428)	0.403 (0.402 - 0.404)
		MCC	0.305 (0.305 - 0.306)	0.306 (0.306 - 0.307)	0.281 (0.279 - 0.282)
		**AUC**	**0.724 (0.724 - 0.725)**	**0.726 (0.726 - 0.726)**	**0.708 (0.704 - 0.711)**
	**6 features (by LASSO)**	Accuracy	60.412% (60.331% - 60.494%)	56.534% (56.263% - 56.806%)	48.953% (48.899% - 49.007%)
		PPV	29.350% (29.323% - 29.376%)	27.992% (27.895% - 28.090%)	25.490% (25.476% - 25.505%)
		Sensitivity	85.079% (84.895% - 85.263%)	89.807% (89.550% - 90.063%)	95.329% (95.283% - 95.375%)
		Specificity	54.991% (54.855% - 55.127%)	49.222% (48.837% - 49.607%)	38.761% (38.685% - 38.837%)
		F1	0.436 (0.436 - 0.437)	0.427 (0.426 - 0.428)	0.402 (0.402 - 0.402)
		MCC	0.308 (0.308 - 0.309)	0.304 (0.303 - 0.305)	0.279 (0.279 - 0.280)
		**AUC**	**0.727 (0.726 - 0.727)**	**0.726 (0.726 - 0.726)**	**0.674 (0.674 - 0.675)**
	**18-features**	Accuracy	61.349% (61.286% - 61.412%)	57.871% (57.834% - 57.908%)	50.821% (50.716% - 50.926%)
		PPV	30.040% (30.006% - 30.074%)	28.763% (28.751% - 28.774%)	26.241% (26.202% - 26.279%)
		Sensitivity	86.177% (86.151% - 86.202%)	90.624% (90.573% - 90.675%)	95.509% (95.480% - 95.537%)
		Specificity	55.893% (55.812% - 55.974%)	50.673% (50.617% - 50.730%)	41.000% (40.865% - 41.134%)
		F1	0.445 (0.445 - 0.446)	0.437 (0.437 - 0.437)	0.412 (0.411 - 0.412)
		MCC	0.324 (0.323 - 0.324)	0.320 (0.320 - 0.320)	0.295 (0.295 - 0.296)
		**AUC**	**0.743 (0.742 - 0.743)**	**0.731 (0.730 - 0.731)**	**0.727 (0.727 - 0.728)**

Note: Please refer to supplementary Table S6 for the definitions of performance metrics. Abbreviation: AUC: receiver operating characteristic curve area under the curve.

Table S8(b). Detailed performance data of the LR prediction models with the ROSE package incorporated

Model	Feature set	Performance metric	Model with 85% sensitivity	Model with 90% sensitivity	Model with 95% sensitivity
**LR (ROSE)**	**6 features (by the proposed DT- based method)**	Accuracy	61.299% (61.277% - 61.320%)	57.252% (57.221% - 57.283%)	48.799% (48.743% - 48.855%)
		PPV	29.844% (29.832% - 29.857%)	28.368% (28.352% - 28.383%)	25.389% (25.367% - 25.410%)
		Sensitivity	84.995% (84.993% - 84.997%)	90.001% (89.999% - 90.003%)	94.999% (94.997% - 95.000%)
		Specificity	56.091% (56.065% - 56.117%)	50.055% (50.017% - 50.093%)	38.646% (38.577% - 38.714%)
		F1	0.442 (0.442 - 0.442)	0.431 (0.431 - 0.432)	0.401 (0.400 - 0.401)
		MCC	0.316 (0.316 - 0.316)	0.311 (0.311 - 0.311)	0.276 (0.275 - 0.276)
		**AUC**	**0.756 (0.756 - 0.756)**	**0.756 (0.756 - 0.756)**	**0.756 (0.756 - 0.756)**
	**6 features (by mRMRe)**	Accuracy	60.373% (60.348% - 60.399%)	56.072% (56.038% - 56.106%)	48.527% (48.488% - 48.566%)
		PPV	29.316% (29.301% - 29.330%)	27.794% (27.778% - 27.811%)	25.287% (25.272% - 25.301%)
		Sensitivity	84.994% (84.990% - 84.998%)	90.002% (90.000% - 90.005%)	94.999% (94.997% - 95.002%)
		Specificity	54.963% (54.932% - 54.994%)	48.616% (48.574% - 48.657%)	38.314% (38.266% - 38.362%)
		F1	0.436 (0.436 - 0.436)	0.425 (0.425 - 0.425)	0.399 (0.399 - 0.400)
		MCC	0.307 (0.307 - 0.308)	0.301 (0.301 - 0.301)	0.274 (0.273 - 0.274)
		**AUC**	**0.751 (0.751 - 0.751)**	**0.751 (0.751 - 0.751)**	**0.751 (0.751 - 0.751)**
	**6 features (by LASSO)**	Accuracy	60.023% (59.997% - 60.049%)	55.725% (55.703% - 55.748%)	48.336% (48.291% - 48.381%)
		PPV	29.120% (29.106% - 29.135%)	27.630% (27.619% - 27.640%)	25.215% (25.198% - 25.232%)
		Sensitivity	84.995% (84.993% - 84.997%)	90.001% (89.999% - 90.003%)	94.998% (94.996% - 94.999%)
		Specificity	54.535% (54.504% - 54.566%)	48.193% (48.165% - 48.221%)	38.081% (38.026% - 38.136%)
		F1	0.434 (0.434 - 0.434)	0.423 (0.423 - 0.423)	0.399 (0.398 - 0.399)
		MCC	0.304 (0.304 - 0.304)	0.298 (0.298 - 0.298)	0.272 (0.272 - 0.273)
		**AUC**	**0.757 (0.757 - 0.757)**	**0.757 (0.757 - 0.757)**	**0.757 (0.757 - 0.757)**
	**18-features**	Accuracy	62.309% (62.281% - 62.338%)	57.936% (57.899% - 57.972%)	49.971% (49.909% - 50.033%)
		PPV	30.444% (30.427% - 30.461%)	28.711% (28.692% - 28.729%)	25.838% (25.813% - 25.862%)
		Sensitivity	84.996% (84.994% - 84.997%)	89.997% (89.996% - 89.998%)	94.998% (94.997% - 94.999%)
		Specificity	57.324% (57.289% - 57.358%)	50.890% (50.845% - 50.934%)	40.076% (40.000% - 40.152%)
		F1	0.448 (0.448 - 0.448)	0.435 (0.435 - 0.436)	0.406 (0.406 - 0.407)
		MCC	0.325 (0.325 - 0.326)	0.317 (0.317 - 0.317)	0.285 (0.285 - 0.286)
		**AUC**	**0.767 (0.767 - 0.767)**	**0.767 (0.767 - 0.767)**	**0.767 (0.767 - 0.767)**

Note: Please refer to supplementary Table S6 for the definitions of performance metrics. Abbreviation: AUC: receiver operating characteristic curve area under the curve.

Table S8(c). Detailed performance data of the DNN prediction models with the ROSE package incorporated

Model	Feature set	Performance metric	Model with 85% sensitivity	Model with 90% sensitivity	Model with 95% sensitivity
**DNN (ROSE)**	**6 features (by the proposed DT- based method)**	Accuracy	61.927% (61.748% - 62.105%)	57.939% (57.746% - 58.132%)	50.205% (50.007% - 50.403%)
		PPV	30.216% (30.112% - 30.320%)	28.714% (28.618% - 28.810%)	25.930% (25.853% - 26.007%)
		Sensitivity	84.995% (84.993% - 84.998%)	90.001% (89.998% - 90.004%)	94.997% (94.996% - 94.999%)
		Specificity	56.857% (56.639% - 57.075%)	50.893% (50.658% - 51.128%)	40.361% (40.119% - 40.603%)
		F1	0.446 (0.445 - 0.447)	0.435 (0.434 - 0.436)	0.407 (0.406 - 0.408)
		MCC	0.322 (0.320 - 0.323)	0.317 (0.315 - 0.319)	0.287 (0.285 - 0.288)
		**AUC**	**0.756 (0.755 - 0.758)**	**0.756 (0.755 - 0.758)**	**0.756 (0.755 - 0.758)**
	**6 features (by mRMRe)**	Accuracy	59.823% (59.023% - 60.622%)	56.278% (55.456% - 57.100%)	49.865% (49.093% - 50.637%)
		PPV	29.040% (28.639% - 29.441%)	27.918% (27.556% - 28.280%)	25.813% (25.533% - 26.092%)
		Sensitivity	84.995% (84.992% - 84.998%)	90.003% (89.999% - 90.007%)	94.999% (94.997% - 95.002%)
		Specificity	54.291% (53.316% - 55.266%)	48.866% (47.864% - 49.869%)	39.946% (39.005% - 40.887%)
		F1	0.433 (0.428 - 0.437)	0.426 (0.422 - 0.430)	0.406 (0.402 - 0.409)
		MCC	0.303 (0.296 - 0.310)	0.303 (0.296 - 0.310)	0.284 (0.278 - 0.290)
		**AUC**	**0.750 (0.745 - 0.754)**	**0.750 (0.745 - 0.754)**	**0.750 (0.745 - 0.754)**
	**6 features (by LASSO)**	Accuracy	60.792% (60.748% - 60.837%)	57.055% (57.025% - 57.086%)	50.191% (50.154% - 50.228%)
		PPV	29.553% (29.528% - 29.579%)	28.270% (28.255% - 28.285%)	25.924% (25.909% - 25.939%)
		Sensitivity	84.997% (84.994% - 85.001%)	90.001% (89.999% - 90.004%)	95.001% (94.998% - 95.005%)
		Specificity	55.473% (55.418% - 55.528%)	49.815% (49.778% - 49.853%)	40.343% (40.298% - 40.388%)
		F1	0.439 (0.438 - 0.439)	0.430 (0.430 - 0.430)	0.407 (0.407 - 0.408)
		MCC	0.311 (0.311 - 0.312)	0.309 (0.309 - 0.310)	0.287 (0.287 - 0.287)
		**AUC**	**0.760 (0.759 - 0.760)**	**0.760 (0.759 - 0.760)**	**0.760 (0.759 - 0.760)**
	**18-features**	Accuracy	62.559% (62.351% - 62.767%)	58.980% (58.730% - 59.229%)	52.757% (52.496% - 53.019%)
		PPV	30.599% (30.476% - 30.721%)	29.254% (29.127% - 29.381%)	26.974% (26.867% - 27.082%)
		Sensitivity	84.995% (84.994% - 84.996%)	89.997% (89.996% - 89.998%)	94.999% (94.998% - 95.000%)
		Specificity	57.629% (57.375% - 57.882%)	52.163% (51.859% - 52.468%)	43.474% (43.155% - 43.794%)
		F1	0.450 (0.449 - 0.451)	0.442 (0.440 - 0.443)	0.420 (0.419 - 0.421)
		MCC	0.328 (0.326 - 0.330)	0.326 (0.324 - 0.328)	0.307 (0.305 - 0.309)
		**AUC**	**0.768 (0.767 - 0.769)**	**0.768 (0.767 - 0.769)**	**0.768 (0.767 - 0.769)**

Note: Please refer to supplementary Table S6 for the definitions of performance metrics. Abbreviation: AUC: receiver operating characteristic curve area under the curve.

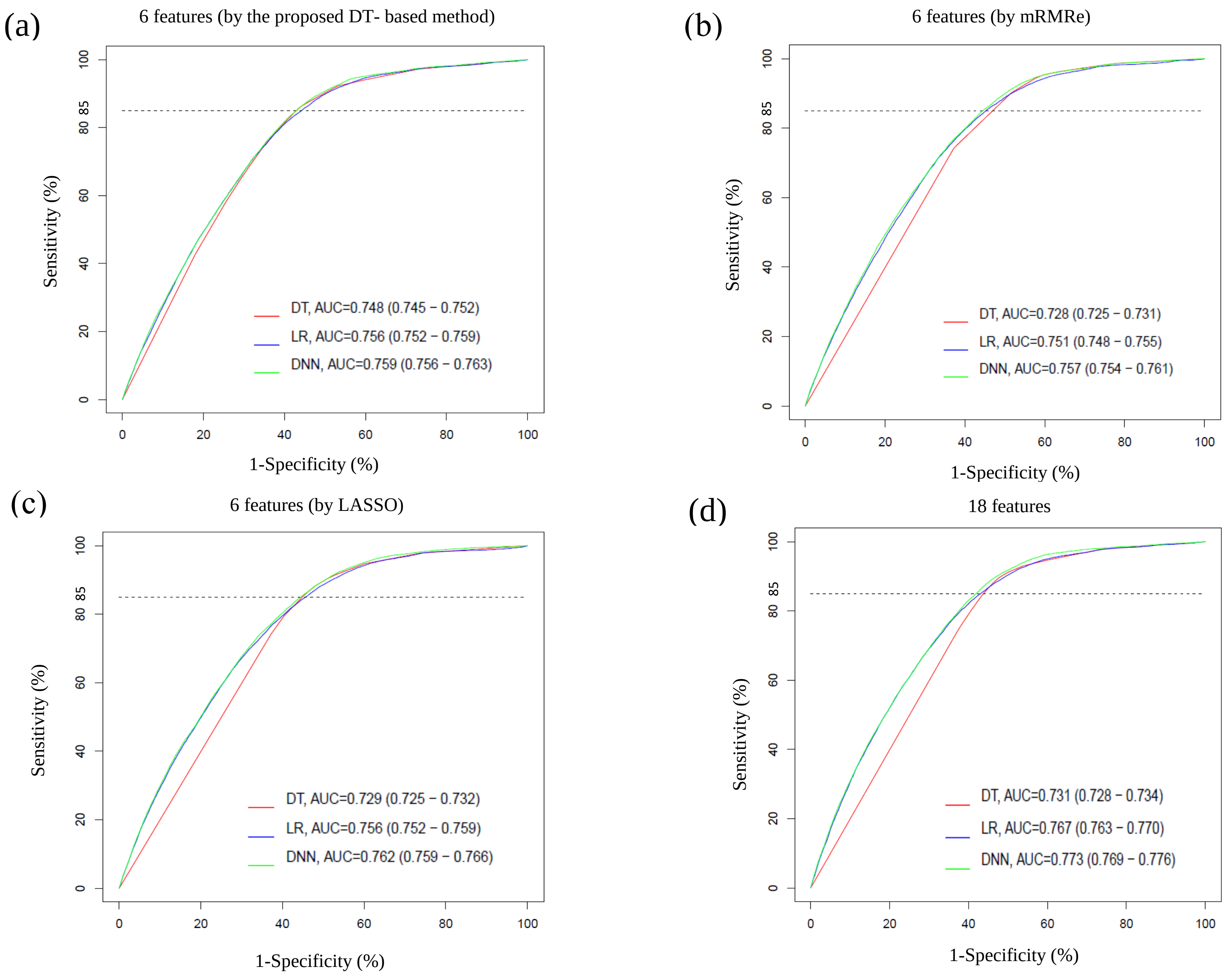

Figure SF1. Receiver operating characteristic (ROC) curves delivered by different types of prediction models with different feature sets. (a) ROC curves with the 6 features identified by the proposed DT based feature selection mechanism. (b) ROC curves with the 6 features identified by the mRMRe package. (c) ROC curves with the 6 features identified by the LASSO package. (d) ROC curves with the 18 features identified in the first-phase of the feature selection process.
